# *Vital Signs:* Clinical Characteristics of Patients with Confirmed Acute Flaccid Myelitis, United States, 2018

**DOI:** 10.15585/mmwr.mm6931e3

**Published:** 2020-08-07

**Authors:** Sarah Kidd, Adriana Lopez, W. Allan Nix, Gloria Anyalechi, Megumi Itoh, Eileen Yee, M. Steven Oberste, Janell Routh

**Affiliations:** ^1^Division of Viral Diseases, National Center for Immunization and Respiratory Diseases, CDC; ^2^Division of STD Prevention, National Center for HIV/AIDS, Viral Hepatitis, STD, and TB Prevention, CDC; ^3^Division of Global HIV and TB, Center for Global Health, CDC.

## Abstract

**Background:**

Acute flaccid myelitis (AFM) is a serious neurologic syndrome that affects mostly children and is characterized by the acute onset of limb weakness or paralysis. Since U.S. surveillance for AFM began in 2014, reported cases have peaked biennially. This report describes the clinical characteristics of AFM patients during 2018, the most recent peak year.

**Methods:**

Medical records from persons meeting AFM clinical criterion (acute onset of flaccid limb weakness) were submitted to CDC. Patients with confirmed AFM met the clinical criterion and had magnetic resonance imaging indicating spinal cord lesions largely restricted to gray matter and spanning one or more vertebral segments. Symptoms, physical findings, test and imaging results, and hospitalization data were abstracted and described.

**Results:**

Among 238 patients with confirmed AFM during 2018, median age was 5.3 years. Among the 238 patients, 205 (86%) had onset during August–November. Most (92%) had prodromal fever, respiratory illness, or both beginning a median of 6 days before weakness onset. In addition to weakness, common symptoms at clinical evaluation were gait difficulty (52%), neck or back pain (47%), fever (35%), and limb pain (34%). Among 211 who were outpatients when weakness began, most (76%) sought medical care within 1 day, and 64% first sought treatment at an emergency department. Overall, 98% of patients were hospitalized, 54% were admitted to an intensive care unit, and 23% required endotracheal intubation and mechanical ventilation.

**Conclusion:**

Clinicians should suspect AFM in children with acute flaccid limb weakness, especially during August–November and when accompanied by neck or back pain and a recent history of febrile respiratory illness. Increasing awareness in frontline settings such as emergency departments should aid rapid recognition and hospitalization for AFM.

*On August 4, 2020, this report was posted online as an *MMWR *Early Release.*

## Introduction

Acute flaccid myelitis (AFM) is a serious neurologic syndrome that can cause paralysis, predominantly in previously healthy children. Similar to poliomyelitis-associated acute flaccid paralysis caused by poliovirus infection, AFM is a syndrome characterized by the acute onset of flaccid limb weakness accompanied by predominantly gray matter lesions in the spinal cord. AFM can progress rapidly over the course of hours or days, leading to permanent paralysis and the life-threatening complication of respiratory failure (*1*).

National surveillance for AFM was initiated in 2014, after California and Colorado reported clusters of AFM or acute limb weakness among previously healthy children, none of whom had laboratory or epidemiologic evidence of poliovirus infection (*2*,*3*). Since 2014, reported AFM cases have peaked in the late summer to early fall every 2 years in the United States (*4*). Although national case reporting for AFM did not begin until 2014, retrospective case investigations have documented sporadic cases before 2014 and increased numbers of cases during 2014, 2016, and 2018 (*5*,*6*). Together, these data suggest that the epidemiology of AFM shifted during or shortly before 2014, and likely reflect a new or emerging etiology.

Multiple viruses, including West Nile virus, adenovirus, and nonpolio enteroviruses, are known to cause AFM in a small percentage of infected persons (*7*–*10*). Pathogens are rarely recovered from the cerebrospinal fluid (CSF) of AFM patients, but enteroviruses are the most common pathogens detected in nonsterile site specimens, such as respiratory and stool specimens (*4*,*11*). Enterovirus D68 (EV-D68) is the most common enterovirus type identified among AFM patients; poliovirus has not been detected in any cases (*4*,*11*,*12*). In addition, recent data, including animal model studies and studies of enterovirus-binding antibodies in CSF, indicate that nonpolio enteroviruses, and EV-D68 in particular, are likely a primary cause of AFM in the United States since 2014 (*13*–*17*). However, other viruses that cause AFM might be contributing to the biennial peaks. A cluster of 11 AFM cases in Colorado associated with enterovirus A71 (EV-A71) contributed to the number of cases reported in 2018 (*18*,*19*).

Based on the observed biennial pattern, another increase in AFM cases is anticipated to occur in the United States in late summer/early fall 2020 (https://www.cdc.gov/grand-rounds/pp/2020/20200703-acute-flaccid-myelitis.html). This report summarizes findings from review of medical records from patients with confirmed AFM in 2018, including cases known to be associated with EV-D68 and EV-A71, and describes the clinical characteristics of patients and settings and timing of seeking medical care for limb weakness. These data might facilitate rapid case recognition of AFM and prompt referral to care.

## Methods

As part of national surveillance, health departments report cases meeting the clinical criterion for AFM (acute onset of flaccid limb weakness) to CDC, along with a patient summary form (completed by health departments) and relevant components of patient medical records when available, including admission and discharge notes, neurology and infectious disease consult notes, laboratory reports, and brain and spine magnetic resonance imaging (MRI) reports. A confirmed AFM case was defined as an illness meeting the clinical criterion with an MRI indicating a spinal cord lesion largely restricted to gray matter and spanning one or more vertebral segments. Confirmed cases were described by month of onset for August 2014–June 2020. In addition, data on the clinical evaluation and hospitalization for limb weakness, including prodromal and initial symptoms at evaluation, neurologic exam findings, laboratory results, MRI findings, and type of medical setting where the patient was evaluated, were abstracted from records of patients with confirmed AFM who had onset of limb weakness during 2018.

Neurologic exam findings were abstracted from the first neurology consultation note following onset of limb weakness or, if that was not available, from the first and most complete documented neurologic exam following the onset of weakness. MRI findings were abstracted from reports of the most abnormal brain and spine MRIs available.

Abstracted laboratory findings included results for enterovirus/rhinovirus (EV/RV)* detection and typing tests performed at external laboratories or CDC’s AFM laboratory. CDC laboratory methods have been described previously (*4*). EV-D68 and EV-A71 cases were defined as a confirmed AFM case with a CSF, respiratory, stool, or serum specimen that tested positive for EV-D68 or EV-A71, respectively, at either an external laboratory or the CDC laboratory. Data on long-term patient outcomes were not available for this analysis.

## Results

Since surveillance for AFM began following the initial clusters reported in 2014, nationwide outbreaks have occurred in 2016 and 2018 (Figure). A total of 238 confirmed AFM cases with onset in 2018 were reported to CDC; among these, onset of limb weakness in 205 (86%) occurred during August–November, including 87 (37%) with onset during September (Figure). Among 219 (92%) patients receiving tests for EV/RV, 107 (49%) had at least one EV/RV-positive specimen (Table 1). A higher proportion of respiratory specimens were positive for EV/RV (48%), compared with other specimen sources (3% [serum] to 20% [stool]). EV-D68 was the most common virus type identified, and most EV-D68 (33 of 34) and EV-A71 (10 of 12) cases were identified from respiratory specimens.

**FIGURE:**
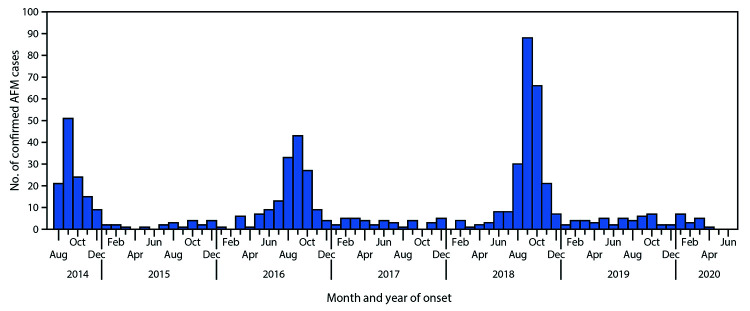
Confirmed cases of acute flaccid myelitis (AFM) reported to CDC (N = 633), by month and year of onset — United States, August 1, 2014– June 30, 2020*^,†^ The figure is a histogram, an epidemiological curve showing 633 confirmed cases of acute flaccid myelitis in the United States reported to CDC United States during August 1, 2014– June 30, 2020, by month and year of onset. * As of July 24, 2020. ^^†^^ Total cases reported each year: 2014 = 120; 2015 = 22; 2016 = 153; 2017 = 38; 2018 = 238; 2019 = 46; 2020 = 16.

**TABLE 1:** Enterovirus/rhinovirus (EV/RV) polymerase chain reaction test results from respiratory, stool, cerebrospinal fluid (CSF), and serum specimens collected from patients with onset of confirmed acute flaccid myelitis (N = 238) — United States, 2018

Specimen source	No. of patients with specimens available (% of 238)	No. (%) positive	EV/RV type results (no.)
Any source*	219 (92)	107 (49)	EV-D68 (34)
			EV-A71 (12)
			Coxsackievirus A2 (1)
			Coxsackievirus A4 (1)
			Coxsackievirus A9 (1)
			Coxsackievirus A16 (1)
			Coxsackievirus B3 (1)
			Echovirus 11 (1)
			RV-A101 (2)
			RV-A24 (1)
			RV-A38 (1)
			RV-A54 (1)
			RV-A81 (1)
			RV-A85 (1)
			RV-B4 (1)
			RV-C54 (1)
			Other/Untyped EV/RV (46)
Respiratory	190 (80)	92 (48)	EV-D68 (33)
			EV-A71 (10)
			RV-A101 (2)
			RV-A24 (1)
			RV-A38 (1)
			RV-A54 (1)
			RV-A81 (1)
			RV-A85 (1)
			RV-B4 (1)
			RV-C54 (1)
			Other/Untyped EV/RV (40)
Stool	119 (50)	24 (20)	EV-D68 (3)
			EV-A71 (2)
			Coxsackievirus A2 (1)
			Coxsackievirus A4 (1)
			Coxsackievirus A9 (1)
			Coxsackievirus A16 (1)
			Coxsackievirus B3 (1)
			Echovirus 11 (1)
			Other/Untyped EV/RV (13)
CSF	187 (79)	9 (5)	EV-D68 (2)
			EV-A71 (1)
			Other/Untyped EV/RV (6)
Serum	90 (38)	3 (3)	EV-D68 (1)
			Echovirus 11 (1)
			Other/Untyped EV/RV (1)

* Some patients had multiple positive specimens.

The median age of patients with confirmed AFM was 5.3 years (range = 0.5–81.8 years), and 58% were male (Table 2). Patients were reported from 42 states throughout the country; 53% identified as white, 20% as Hispanic, and 9% as black. A prodromal illness preceding the onset of limb weakness was documented in most (97%) patients, with respiratory illness (80%) and fever (77%) the most commonly reported prodromal signs and symptoms. Many patients also had documented neck or back pain (46%) or headache (37%) preceding the onset of limb weakness. Among those who reported prodromal symptoms, the onset of respiratory illness generally occurred earlier than the onset of fever, headache, or neck or back pain.

**TABLE 2:** Demographic and clinical characteristics of patients with onset of confirmed acute flaccid myelitis (N = 238), by virus type — United States, 2018

Characteristic	No. (%)			
	EV-D68 (n = 34)	EV-A71 (n = 12)	Other (n = 192)*	Total (N = 238)
**Demographic**				
Median age, yrs	5.9	1.6	5.3	5.3
Range	1.4–56.9	0.9–32.7	0.5–81.8	0.5–81.8
IQR	3.7–7.9	1.1–2.1	3.4–8.3	3.3–8.2
Male sex	20 (59)	11 (92)	107 (56)	138 (58)
**Geographic region**				
South	16 (47)	0 (0)	64 (33)	80 (34)
Midwest	6 (18)	1 (8)	54 (28)	61 (26)
West	6 (18)	11 (92)	39 (20)	56 (24)
Northeast	6 (18)	0 (0)	35 (18)	41 (17)
**Race/Ethnicity**				
White	23 (68)	10 (83)	92 (48)	125 (53)
Hispanic	5 (15)	1 (8)	41 (21)	47 (20)
Black	2 (6)	0 (0)	19 (10)	21 (9)
Asian	1 (3)	0 (0)	7 (4)	8 (3)
Multiracial	0 (0)	0 (0)	4 (2)	4 (2)
Native Hawaiian/Pacific Islander	0 (0)	0 (0)	1 (1)	1 (0)
Unknown	3 (9)	1 (8)	28 (15)	32 (13)
**Prodromal signs/symptoms in the 4 weeks before onset of limb weakness**				
Any illness, no. (% of total)	34 (100)	12 (100)	184 (96)	230 (97)
Days before weakness onset (IQR)^†^	5 (4–7)	3 (2.5–6.5)	6 (3–9)	6 (3–9)
Any respiratory illness or fever, no. (% of total)	34 (100)	12 (100)	174 (91)	220 (92)
Days before weakness onset (IQR)^†^	5 (4–7)	3 (2–5)	6 (3–9)	6 (3–8)
Any respiratory illness, no. (% of total)	33 (97)	7 (58)	151 (79)	191 (80)
Days before weakness onset (IQR)^†^	5 (4–7)	3 (1–5)	6 (4–9)	6 (4–9)
Any fever, no. (% of total)	28 (82)	12 (100)	144 (75)	184 (77)
Days before weakness onset (IQR)^†^	3 (2–6)	3 (2–5)	3 (2–6)	3 (2–6)
Neck/back pain, no. (% of total)	18 (53)	4 (33)	88 (46)	110 (46)
Days before weakness onset (IQR)^†^	2 (1–3)	1.5 (0.5–4)	1 (1–3)	1.5 (1–3)
Headache, no. (% of total)	12 (35)	2 (17)	73 (38)	87 (37)
Days before weakness onset (IQR)^†^	3 (2–3)	2 (2–2)	2 (1–6)	2 (1–5.5)
Any gastrointestinal illness, no. (% of total)	9 (26)	6 (50)	38 (20)	53 (22)
Days before weakness onset (IQR)^†^	2 (1–4)	2.5 (2–3)	3 (2–4)	2 (2–4)
Rash, no. (% of total)	3 (9)	7 (58)	13 (7)	23 (10)
Days before weakness onset (IQR)^†^	4.5 (2–7)	4 (3–6)	9.5 (3–18.5)	4 (3–7)
**Signs/Symptoms at evaluation**				
Gait difficulty	19 (56)	6 (50)	99 (52)	124 (52)
Pain in neck or back	20 (59)	4 (33)	87 (45)	111 (47)
Fever	16 (47)	8 (67)	59 (31)	83 (35)
Pain in affected limb(s)	10 (29)	3 (25)	69 (36)	82 (34)
Headache	13 (38)	2 (17)	51 (27)	66 (28)
Neck weakness	7 (21)	1 (8)	31 (16)	39 (16)
Ataxia/Discoordination	6 (18)	8 (67)	24 (13)	38 (16)
Facial weakness	9 (26)	0 (0)	28 (15)	37 (16)
Dysphagia	6 (18)	2 (17)	23 (12)	31 (13)
Bladder retention/incontinence	2 (6)	3 (25)	23 (12)	28 (12)
Altered consciousness	2 (6)	6 (50)	18 (9)	26 (11)
Dysarthria	6 (18)	1 (8)	17 (9)	24 (10)
Numbness in affected limb(s)	2 (6)	1 (8)	13 (7)	16 (7)
Paresthesia in affected limb(s)	0 (0)	0 (0)	16 (8)	16 (7)
Bowel retention/incontinence	1 (3)	3 (25)	10 (5)	14 (6)
Diplopia	2 (6)	1 (8)	9 (5)	12 (5)
Ptosis	3 (9)	0 (0)	6 (3)	9 (4)
Seizures	2 (6)	1 (8)	3 (2)	6 (3)
**Initial neurologic exam findings**				
Decreased strength in upper limb(s)	22 (65)	4 (33)	127 (66)	153 (64)
Decreased strength in lower limb(s)	16 (47)	2 (17)	67 (35)	85 (36)
Any sensory abnormalities	5 (15)	0 (0)	25 (13)	30 (13)
Any cranial nerve abnormalities	12 (35)	2 (17)	37 (19)	51 (21)
Abnormal mental status	1 (3)	5 (42)	6 (3)	12 (5)
**CSF microscopic examination^§^**				
CSF pleocytosis	31/32 (97)	12/12 (100)	140/166 (84)	183/210 (87)
Median cells/mm^3^	95	125	91.5	94
Range	9–499	17–685	6–814	6–814
IQR	36–170	67–480	42.5–157.5	43–163
**MRI findings^¶^**				
Supratentorial lesions	3/33 (9)	3/12 (25)	23/182 (13)	29/227 (13)
Brainstem lesions	15/33 (45)	11/12 (92)	74/182 (41)	100/227 (44)
Cerebellar lesions	1/33 (9)	9/12 (75)	38/182 (21)	50/227 (22)
Cervical cord lesions	33/34 (97)	12/12 (100)	174/187 (80)	219/233 (94)
Thoracic cord lesions	28/31 (90)	6/11 (55)	142/163 (87)	176/205 (86)
Conus lesions	16/25 (64)	2/5 (40)	2/5 (36)	71/177 (40)
Nerve root enhancement	5/33 (15)	6/12 (50)	38/182 (21)	49/227 (22)

**Abbreviations:** CSF = cerebrospinal fluid; EV = enterovirus; IQR = interquartile range; MRI = magnetic resonance imaging.

* Other category includes all patients who did not have a positive EV-D68 or EV-A71 test, including those who were never tested, those who had negative test results, and those who had positive test results for other viruses.

^†^ Timing calculated among cases with the prodromal illness/symptom and documented dates of onset.

^§^ Among 210 cases with CSF results available. Median cells/mm^3^ calculated among cases with CSF pleocytosis (>5 white blood cells per mm^3^).

^¶^ Supratentorial, brainstem, and cerebellar lesions among 227 cases with brain MRI reports available. Cervical cord lesions among 233 cases with cervical spine reports available, thoracic cord lesions among 205 cases with thoracic spine reports available, and conus lesions among 177 cases with lumbosacral spine reports available. Nerve root enhancement among 227 cases with a contrast MRI of the spine.

At the time that patients were seen at a hospital for weakness, the most commonly documented symptoms were gait difficulty (52%), neck or back pain (47%), fever (35%), and pain in the affected limb or limbs (34%). Upper extremity weakness (64%) was more commonly noted on initial neurologic exam than was lower extremity weakness (36%). Only 13% of patients had documented sensory abnormalities; 21% had cranial nerve abnormalities, and 5% had altered mental status on exam.

All patients with confirmed AFM had, by definition, at least one abnormal spinal cord MRI indicating predominantly gray matter lesions. Based on medical records received at CDC, 227 (95%) patients had a brain MRI, 233 (98%) had a cervical spine MRI, 205 (86%) had a thoracic spine MRI, and 177 (74%) had a lumbosacral spine MRI performed. Among those receiving MRIs, cervical cord lesions were most commonly observed (219 of 233; 94%), followed by thoracic cord lesions (176 of 205; 86%). Although fewer patients had a lumbosacral spine MRI, conus lesions were seen in 40% (71 of 177) of those with MRIs. Brainstem lesions were observed in 44% (100 of 227) of those who received a brain MRI.

Most (233 of 238; 98%) patients were hospitalized (Table 3). Twenty-five patients (11%) were hospitalized at least 1 day before onset of limb weakness and developed weakness while inpatients, 206 (87%) were hospitalized on or after the day limb weakness began, and the date of onset for two patients was unclear. Overall, 54% of all patients were admitted to an intensive care unit, and 23% required intubation and mechanical ventilation. Steroids and intravenous immunoglobulin (IVIG) were the commonly administered treatments (Table 3).

**TABLE 3:** Characteristics of hospitalization, treatment, and first medical encounter after onset of limb weakness among patients with confirmed acute flaccid myelitis (N = 238), by virus type — United States, 2018

Characteristic	No. (%)			
	EV-D68 (N = 34)	EV-A71 (N = 12)	Other (N = 192)*	Total (N = 238)
**Hospitalization**				
Hospitalized	34 (100)	12 (100)	187 (97)	233 (98)
Hospitalized ≥1 day before onset of limb weakness	7 (21)	4 (33)	14 (7)	25 (11)
Hospitalized on same day or after onset of limb weakness	27 (79)	8 (67)	171 (89)	206 (87)
Hospitalized, unknown timing	0 (—)	0 (—)	2 (1)	2 (1)
**Timing from onset of weakness to hospitalization,^†^ days**				
Median interval from onset of weakness to hospitalization	1	0.5	1	1
Range	0–5	0–5	0–54	0–54
IQR	0–2	0–2.5	0–2	0–2
0–1	20/27 (74)	5/8 (63)	109/171 (64)	134/206 (65)
2–3	6/27 (22)	2/8 (25)	44/171 (26)	52/206 (25)
4–7	1/27 (4)	1/8 (13)	8/171 (5)	10/206 (5)
>7	0/27 (—)	0/8 (—)	10/171 (6)	10/206 (5)
**Treatment received**				
Steroids, no IVIG	8 (24)	1 (8)	46 (24)	55 (23)
IVIG, no steroids	9 (26)	8 (67)	37 (19)	54 (23)
Both steroids and IVIG	12 (35)	2 (17)	67 (35)	81 (34)
Plasma exchange	5 (15)	1 (8)	26 (14)	32 (13)
Admitted to ICU	25 (74)	5 (42)	99 (52)	129 (54)
Respiratory support	19 (56)	3 (25)	43 (22)	65 (27)
Mechanical ventilation	15 (44)	1 (8)	39 (20)	55 (23)
**Location of first medical encounter after onset of weakness^§^**				
Emergency department	17/27 (63)	2/8 (25)	115/176 (65)	134/211 (64)
Primary care provider	5/27 (19)	4/8 (50)	40/176 (23)	49/211 (23)
Urgent care provider	4/27 (15)	0/8 (—)	12/176 (7)	16/211 (8)
Unknown/missing/other	1/27 (4)	2/8 (25)	9/176 (5)	12/211 (6)
**Timing from onset of limb weakness to first medical encounter,^§^ days**				
Median interval from onset of weakness to first medical encounter^¶^	0	0	1	0
Range	0–3	0–1	0–15	0–15
IQR	0–1	0–0.5	0–1	0–1
0–1	19/27 (70)	8/8 (100)	133/176 (76)	160/211 (76)
2–3	5/27 (19)	0/8 (—)	29/176 (16)	34/211 (16)
4–7	0/27 (—)	0/8 (—)	4/176 (2)	4/211 (2)
>7	0/27 (—)	0/8 (—)	2/176 (1)	2/211 (1)
Unknown/Missing	3/27 (11)	0/8 (—)	8/176 (5)	11/211 (5)

**Abbreviations:** EV = enterovirus; ICU = intensive care unit; IQR = interquartile range; IVIG = intravenous immunoglobulin.

* Other category includes all patients who did not have positive EV-D68 or EV-A71 test results, including those who were never tested, those who had negative test results, and those who had positive test results for other viruses.

^†^ Among the 206 patients (EV-D68 = 27; EV-A71 = 8; other = 171) who were hospitalized on or after the date of onset of limb weakness;

^§^ Among the 211 patients who had onset of limb weakness as an outpatient (206 patients who were hospitalized on or after the date of onset of limb weakness and five patients who were never hospitalized).

^¶^ Among 200 patients who had onset of limb weakness as an outpatient and known timing of onset of limb weakness and first medical encounter (211 patients minus the 11 with timing unknown).

Among the 211 patients who developed limb weakness as an outpatient (including five who were never hospitalized), most (134 of 211; 64%) initially sought treatment at an emergency department and 49 (23%) at a primary care provider. Most (160 of 211; 76%) sought medical care within 1 day of onset of limb weakness. Similarly, among patients hospitalized on the same day or after onset of weakness, most (134 of 206; 65%) were hospitalized within 1 day of onset.

When EV-D68 and EV-A71 cases were analyzed, patients with known EV-D68 infection were older (median age = 5.9 years) than were those with EV-A71 infection (median age = 1.6 years). In addition, patients with EV-D68 were reported from across the country, whereas 11 of 12 patients with EV-A71 were geographically and temporally clustered in Colorado (Table 2). All EV-D68 and EV-A71 cases had a prodromal illness before onset of limb weakness. Prodromal respiratory illness was more common among EV-D68 (97%) than among EV-A71 cases (58%). Prodromal rash was more common among EV-A71 (58%) than among EV-D68 cases (9%). The most common signs and symptoms accompanying limb weakness among EV-D68 cases were neck or back pain (59%), gait difficulty (56%), and fever (47%), whereas among EV-A71 cases, the most common signs and symptoms were fever (67%), ataxia (67%), gait difficulty (50%), and altered consciousness (50%). Abnormal findings on brain MRI were less common among EV-D68 than among EV-A71 cases, but a higher proportion of EV-D68 than EV-A71 cases were admitted to an intensive care unit and required intubation and mechanical ventilation (Table 3).

## Discussion

The findings in this report are consistent with, and build upon, previous reports describing patients with confirmed AFM during 2018 and earlier peak years (*4*,*11*,*12*,*19*). The current analysis supports previous reports documenting the frequent presence of respiratory symptoms, fever, or both before the onset of limb weakness and a predominance of upper limb involvement among AFM patients. In addition, prodromal neck or back pain or headache before onset of limb weakness were identified and described in the present cohort. Eleven percent of AFM patients were hospitalized at least 1 day before the onset of limb weakness, indicating that prodromal symptoms might be severe in some patients.

Evaluating a child with weakness and differentiating AFM from other causes of weakness can be challenging. In younger children especially, weakness might manifest as decreased use of a limb, which might mistakenly be attributed to musculoskeletal pain or injury. Gait difficulty, neck or back pain, fever, limb pain, and headache were often present when AFM patients sought care for limb weakness. The presence of these or any neurologic signs or symptoms in a child with acute limb weakness or decreased use of an extremity should heighten clinical suspicion of AFM, particularly in the setting of a recent respiratory or febrile illness.

These findings also indicate that clinical characteristics of AFM patients might differ by viral etiology. However, these results should be interpreted with caution because 11 of the 12 EV-A71 cases were reported from a single state (Colorado), potentially influencing the description of EV-A71 cases. In addition, these results could be affected by biased ascertainment of viral infection if cases with certain characteristics (e.g., severe respiratory symptoms) were more likely to be tested early in the course of illness, when testing is more likely to yield a pathogen (*4*,*11*). Some actual EV-D68 and EV-A71 cases likely did not have virus detected and were misclassified in the “other” category. However, these findings are consistent with other comparisons of EV-D68– and EV-A71–associated AFM cases, including those that compared cases within the same institution (*18*). Although there is considerable overlap in symptoms and findings associated with these two viruses, different viruses are likely associated with different AFM phenotypes.

Regardless of etiology, patients generally sought medical attention soon after onset of limb weakness; 76% of those with onset as an outpatient sought medical care within 1 day of onset of weakness, and most were initially evaluated in an emergency department. Because AFM can progress rapidly and lead to respiratory failure requiring intubation and mechanical ventilation, patients with suspected AFM should be immediately hospitalized and monitored for respiratory deterioration. Hospitalization also facilitates evaluation, including consultation with specialists and MRI of the brain and spine. Most patients were hospitalized within 1 day of onset of weakness, but 10% were not hospitalized until ≥4 days after onset of limb weakness, perhaps indicating delays in recognition and an opportunity for improvement.

The findings in the report are subject to at least two limitations. First, this study was restricted to cases reported to CDC, which likely underestimate the actual number of AFM cases owing to underascertainment of AFM and underreporting of cases to health departments. Second, the analysis was limited to medical record abstraction data and, in many cases, to data from the early course of hospitalization. Although data on long-term outcomes were not available for this analysis, these data are now being collected and will be the subject of future reports.

Despite these limitations, the data in this report further elucidate the clinical characteristics of AFM and should aid recognition of signs and symptoms, subsequent evaluation, and referral to care. Early recognition of AFM is important for clinical management and for specimen collection and detection of the underlying etiology. AFM should be suspected in any child with acute flaccid limb weakness. Onset during the months of August–November of peak years, history of recent febrile respiratory illness, and presence of neck or back pain or any neurologic symptom should raise suspicion for AFM.

Based on recent trends, another peak AFM year is anticipated in 2020. It is not known whether or how the COVID-19 pandemic and recommended social distancing measures will affect enterovirus circulation or trends in AFM. COVID-19’s impact on the health care system and health care–seeking behaviors will likely present additional challenges to the recognition and evaluation of patients with AFM. Non-COVID-19 emergency department visits declined in 2020 (*20*), and the pandemic could possibly contribute to delays in care or to an increased proportion of clinical evaluations taking place via telephone or telemedicine. During this time, it will be critical for parents and clinicians to be aware of signs and symptoms suggestive of AFM and maintain vigilance for this condition during 2020.

**Summary**

**What is already known about this topic?**

Since U.S. surveillance for acute flaccid myelitis (AFM) began in 2014, reported cases have peaked biennially. Most cases occur in children during late summer and early fall.

**What is added by this report?**

Among 238 patients with confirmed AFM during 2018, most (92%) had prodromal fever, respiratory illness, or both. In addition to weakness, common symptoms were gait difficulty (52%), neck or back pain (47%), fever (35%), and limb pain (34%). Among 211 who were outpatients when weakness began, 64% sought treatment at an emergency department. Overall, 23% required endotracheal intubation and mechanical ventilation.

**What are the implications for public health practice?**

Clinicians should suspect AFM in children with acute flaccid limb weakness, especially when accompanied by neck or back pain and a recent history of febrile respiratory illness. Increasing awareness in frontline settings such as emergency departments should aid rapid recognition and hospitalization for AFM.

## Footnotes

* Enteroviruses and rhinoviruses are closely related picornaviruses. Most widely available polymerase chain reaction tests for enterovirus amplify a viral gene sequence that is highly conserved and is similar between enteroviruses and rhinoviruses. Therefore, these tests do not distinguish between enteroviruses or rhinoviruses, and additional testing is needed to differentiate between these groups of viruses.
